# Over the Daisies

**Published:** 1887-10

**Authors:** 


### OVER THE DAISIES.

Now, alas ! the meadow daisies,
Chill and broken, helpless lie,
Holding still, their pallid faces,
Upward to the pitying sky.

How they loved the flowing river,
And the brooklets mossy side,
Dotting o'er in gold and silver,
Hill and valley, far and wide.

Oh, ye visions of the summer,
When the winter frosts and chill,
Have ye yet some bright hereafter,
Of the spirit, onward still ?

Yes, I seem to feel it, know it,
All of beauty all of love,
Song of bird or tender floweret,
Hath its counterpart above.

De Travers.

